# Pre-clinical efficacy of CD20-targeted chimeric antigen receptor T cells for non-Hodgkin's lymphoma

**DOI:** 10.1007/s12672-022-00588-w

**Published:** 2022-11-09

**Authors:** Hairuo Wen, Xiaoyan Lou, Zhe Qu, Chao Qin, Hua Jiang, Ying Yang, Liqing Kang, Xingchao Geng, Lei Yu, Ying Huang

**Affiliations:** 1 Key Laboratory of Beijing for Safety Evaluation of Drugs, National Center for Safety Evaluation of Drugs, National Institutes for Food and Drug Control, Beijing, 100176 People’s Republic of China; 2 Shanghai Unicar-Therapy Bio-Medicine Technology Co., Ltd, No 1525 Minqiang Road, Shanghai, People’s Republic of China

**Keywords:** CD20 targeted, CAR-T, Efficacy, Non-Hodgkin's lymphoma, NSG mice, Cytokine

## Abstract

**Background:**

A 4**-**1BB/CD3-ζ-costimulated CAR-T against CD20 (CAR-T20) was subjected to a systemic efficacy evaluation in a cell co-culture model, and NOD-SCID IL-2 receptor gamma null mice (short for NSG mice) were xenografted with human Burkitt's lymphoma Raji cells.

**Methods:**

CAR-T20 cells were incubated with target cells (K562, K562 CD20 or Raji cells) at ratios of 10:1 and 5:1 for 24 h, and the killing rate was estimated by an LDH cytotoxicity assay. To evaluate the effect of CAR-T20 on the survival time of tumor-bearing animals, 30 NSG mice were employed, and Raji-Luc cells (5 × 10^5^ cells per mouse) were administered prior to CAR-T20 administration. The survival time, optical intensity of Raji-Luc cells, clinical symptoms, and body mass of the animals were observed. Another 144 male NSG mice were employed to investigate the proliferation and antitumor effects of CAR-T20. Human cytokine and murine cytokines were detected at 1, 7, 14, 21, 28, 42, 56 and 90 days post-CAR-T administration, while biochemistry index analysis, T-cell and CAR-T-cell detection in peripheral blood, and histopathological examination were performed at 14, 28, 56 and 90 days post-administration.

**Results:**

CAR-T20 cells had a specific killing effect on CD20-expressing cells in vitro. At a dose of 1 × 10^6^ per mouse or above, CAR-T20 prolonged the median survival time from 14 days to more than 3 months, inhibited the proliferation of Raji cells in mice, and alleviated the clinical manifestations and weight loss caused by the Raji-Luc cell load. CAR-T20 at a dose of 2 × 10^6^ per mouse or above inhibited the proliferation of Raji cells in mice for up to 111 days post-administration without recurrence. The numbers of T cells and CAR-T cells in the animals administered CAR-T20 increased significantly when Raji cells were markedly proliferated and subsequently decreased when Raji cells were predominantly inhibited. CAR-T20 increased human IFN-γ, murine TNF and murine IL-6 levels and decreased human IL-10 levels in tumor-bearing mice. The incidences of xenografted tumors in organs/tissues were also reduced effectively by CAR-T20.

**Conclusion:**

The effective dose of CAR-T20 in mice starts from 1 × 10^6^ per mouse, equivalent to a clinical dose of 5 × 10^6^/kg. Together, our data support the clinical translation of CAR-T20 for R/R B-cell NHL patients.

**Supplementary Information:**

The online version contains supplementary material available at 10.1007/s12672-022-00588-w.

## Background

Non-Hodgkin's lymphoma (NHL) is one of the most common hematological malignancies originating from lymph nodes and/or other extra nodal lymphoid tissues, of which 60 to 70% are B-cell-derived lymphomas. As reported, in 2020, there were more than 500,000 cases of non-Hodgkin lymphoma worldwide and more than 250,000 deaths [1]. The clinical uses of anti-CD20 monoclonal antibodies (such as rituximab [2]), anti-CD20 and CD3 bi-specific antibodies (such as pcritamab [3] and mosunetuzumab [4]), antibody drug conjugates (such as CD19 antibody conjugated to pyrrolobenzene and diazepine dimer cytotoxin and loncastuximab tesirine [5]), and immune checkpoint inhibitors (such as penpulimab [6]), have greatly improved the treatment of NHL. However, more than 40% of patients develop primary drug resistance or relapse after current first-line drug treatment and the prognosis after relapse is poor, with a median survival of only half a year [7]. The standard treatment for relapsed/refractory B-cell NHL is chemotherapy combined with autologous stem cell transplantation; however nearly 50% of patients cannot be treated due to poor physical condition, and the relapse rate after treatment is nearly 50% [8].

Chimeric antigen receptor T (CAR-T) cell immunotherapy as a novel precise targeted therapy for tumor treatment has made remarkable achievements in leukemia. At present, five CAR-T-cell therapy products targeting CD19 and two targeting B-cell maturation antigen (BCMA) have been approved by the U.S. Food and Drug Administration (FDA) for marketing. The related indications include: treatment of recurrent/refractory diffuse large B-cell lymphoma, follicular lymphoma, mononuclear cell leukemia and multiple myeloma. The complete remission rate and overall survival of patients receiving CAR-T therapy were significantly improved [9], and it has become the most promising treatment option for hematological malignancies [10]. Through genetic modification of T cells in the peripheral blood of patients or donors, this therapy enables T cells to bind and activate specific antigens on the surface of tumor cells, and directly kill tumor cells by releasing perforin, clindamycin B, *etc*. [11]. Significant efficacy has been achieved in the treatment of Hodgkin lymphoma. For instance, liso-cel achieved the best overall response rate of 80% and the best complete response rate of 59% [12]. Although CD19-targeting CAR-T products have been approved and marketed, the overall response rate is only 52–93% [13–16], still most patients fail to benefit from the first use of CD19-targeting CAR-T treatment. In addition, the 12-month progression-free survival rate of the marketed product is only 44%-65%, indicating that patients will relapse quickly even if remission was achieved [13–16]. Among them, 30% of recurrent patients show no or reduced expression of CD19 targets (antigen relapse) [17], which makes CD19-CAR-T lose the chance of retreatment, and there is no new effective treatment method at present.

**Fig. 1:**
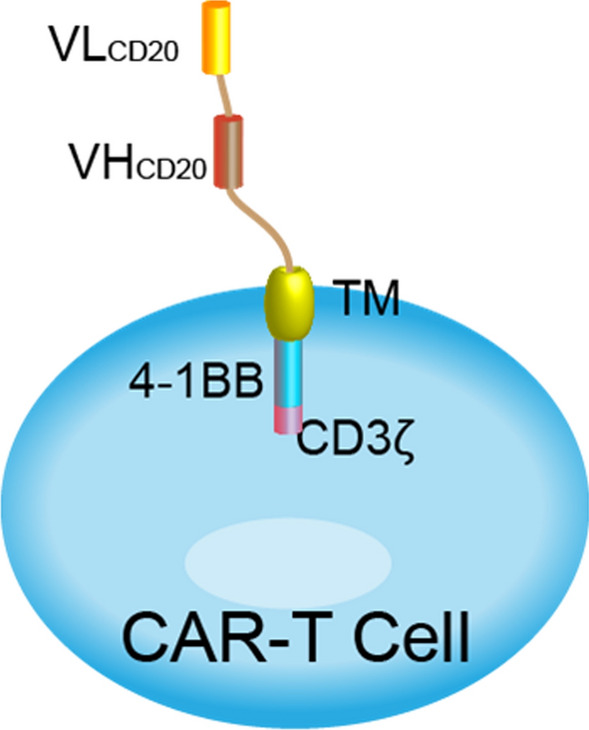
Structure and characteristics of CAR-T20. CAR-20 is a 4**-**1BB/CD3-ζ-costimulated CAR-T targeted at CD20, and it is modified by LV2159K lentiviral vector transduction

Given the long-term success of anti-CD20 monoclonal antibodies in the treatment of B-cell NHL, CD20 is considered an ideal target for the design of CARs. CD20 is an important marker of peripheral B cells. CD20 is expressed in more than 95% of b-cell lymphomas, including diffuse large b-cell lymphoma, follicular lymphoma, classical Hodgkin's lymphoma, extranodal marginal zone lymphoma of muco-associated lymphoid tissue, mantle cell lymphoma, *etc*. [18, 19], and is expressed only in B cells. Compared with CD19, the rate of endocytosis of CD20 antigen after antibody binding is much slower [20]. This antigenic stability could theoretically have a positive impact on the quality of immune synapses, resulting in more robust CAR triggering and t-cell activation effects [12, 21]. As reported by Till et al., CD20-specific CAR-T-cells have antitumor potential in patients with relapsed or refractory indolent B-cell lymphoma [22]. The addition of 4–1BB, as a co-stimulatory, led to an overall response rate of 80–83% and a complete response rate of 17–50%, respectively [23]. In this study, a 4**-**1BB/CD3-ζ-costimulated CAR-T against CD20 (CAR-T20) was developed (Fig. 1), and the efficacy of CAR-T20 was investigated using an in vitro cell co-culture model as well as a NOD-*Prkdc*^*scid*^*IL2rg*^*tm1*^/Bcgen (NSG) mouse model xenografted with human Burkitt's lymphoma Raji cells. This study provides comprehensive efficacy data for CAR-T20 cells and supports its Investigational New Drug application for clinical trial permission in China.

## Materials and methods

### Generation of CAR-T20

The antigen-binding domain of the CD20 CAR consists of a scFv derived from the Leu16 mAb, the co-stimulatory 4-1BB domain, and the CD3 zeta domain. The CAR construct was co-transfected with 3 packaging plasmids into HEK 293 T packaging cells, and the resulting lenti-viruses were isolated, purified and stored at − 80 °C. Peripheral blood mononuclear cells (PBMCs) were obtained from healthy donors by gradient centrifugation using Lymphoprep (Oriental Hua Hui, Beijing, China). For CAR-T20 cell preparation, T lymphocytes were purified using anti-CD3 positive-selection beads (Miltenyi Bio tec, Bergisch-Gladbach, Germany) and stimulated with anti-CD3/CD28 monoclonal antibodies (MiltenyiBiotec). The T cells were subsequently transduced with recombinant lenti-viral vectors. The CAR-T20 cells were cultured for 12 to 14 days in AIM-V medium (Gibco, NY, USA) supplemented with 10% autologous human serum, 100 IU/mL recombinant human IL-2 (PeproTech, Rocky Hill, USA).

### CAR-T cells and target cells

CAR-T20 cells against CD20 were generated as follows: T cells obtained from leukapheresis products were isolated and stimulated with monoclonal anti-CD3/CD20 antibodies (Miltenyi Biotec, Bergisch-Gladbach, Germany). Subsequent to the primary activation, a lentivirus encoding the CD20-4-1BB-CD3 ζ transgene was transduced to the T cells. The cells were cultured for 12 days in AIM-V media (Gibco, NY, USA) supplemented with 10% autologous serum, 100 IU/ml IL-2, 5 ng/ml IL-7, and 5 ng/ml IL-15.

K562 cell line originated from the human leukemia cell line was purchased from ATCC (Manassas, VA, USA), and the K562 CD20 cell line was constructed by transfection with a antigen CD20 construct using a lentiviral vector (Shanghai Unicar-Therapy Bio-medicine Technology Co.,Ltd., Shanghai, China). Human B-cell lymphoma Raji cell line transduced with firefly luciferase [24] (Raji-Luc) expressing CD20 (expression rate over 99% as examined by flow cytometry) for tumor xenografting was a gift of Professor Weijin Huang at the National Institutes for Food and Drug Control. Cells were cultured using RPMI 1640 medium with 10% fetal bovine serum (Gibco) and 1% penicillin–streptomycin solution at 37 ℃ supplemented with 5% CO_2_. Raji-Luc cells were resuspended in 0.9% NaCl at a concentration of 2.5 × 10^6^ cells/mL for in vivo studies [25].

### Co-culture assay

A lactate dehydrogenase (LDH) cytotoxicity assay was performed to determine the cytotoxicity of anti-CD20 CAR-T cells. Effector cells (CAR-T20 cells) and target cells (K562, K562-CD20 or Raji cells) were co-cultured at effector: target (E:T) ratios of 10:1 and 5:1. The cells were treated in triplicate wells of a 96-well plate in AIM-V medium with 4% fetal bovine serum (Gibco) for 16 ~ 24 h, and the supernatant was collected to measure the amount of LDH released. Cytotoxicity was measured using the Cytotoxicity Detection Kit (Promega) according to the manufacturer’s protocol. The level of LDH was detected at 490 nm using a Multiskan GO spectrophotometer (Thermo Fisher Scientific), and cytotoxicity was calculated as follows: %Lysis = [LDH release_(experimental)_—LDH release_(spontaneous)_]/[LDH release_(maximum)_—LDH release_(spontaneous)_) × 100.

### Animals

In compliance with the IACUC Constitution of NCSED, animal experimental procedures of this study were reviewed and approved by the Institutional Animal Care and Use Committee (IACUC) of the National Center for Safety Evaluation of Drugs (NCSED, IACUC approval No: IACUC- 2021-053, IACUC- 2021-060), in compliance with the IACUC Constitution of NCSED.

Specific pathogen-free NSG (NOD-*Prkdc*^*scid*^*IL2rg*^*tm1*^/Bcgen) mice at 6 week of age were provided by Beijing Biocytogen Co., Ltd (Beijing, China; Animal Certificate No: SCXK (SU) 2016–0004). The mice were kept in individually ventilated cages at a density of 2 ~ 3 animals per cage in a barrier system at 20 ~ 26 °C, with 40 ~ 70% relative humidity, and a 12 h light–dark cycle. Animals had ad libitum access to feed and sterilized tap water via water bottles and were quarantined for at least 6 days before randomization.

### Determination on survival time and *tumor clearance*

To evaluate the effect of CAR-T20 on the survival time of tumor-bearing animals, 30 male NSG mice were employed and administered Raji-Luc cells (5 × 10^5^ cells per mouse) prior to CAR-T20 dosing. These animals were randomized into 5 groups (buffer group, T cell group, CAR-T20 1 × 10^6^ group, CAR-T20 2 × 10^6^ group and CAR-T20 5 × 10^6^ group, 6 in each group) based on their bioluminescence intensities, and animals displaying bioluminescence values less than 3.5 × 10^6^ p/sec/cm^2^/sr (approximately 2 times the background data) were not included. Approximately 96 h after xenografting, animals were administered with buffer (containing human albumin, dextran 40 glucose injection, dimethyl sulfoxide (5%, v/v), glucose sodium chloride injection, and compound electrolyte injection), T cells (10 × 10^6^) and CAR-T20 cells (at dosages of 1 × 10^6^, 2 × 10^6^ and 5 × 10^6^). The dosages of regular CAR-T20 cells were calculated refer to the proportion of CAR^+^ T cells, which was 49.79%, in the batch used. The T-cell dosage was equivalent to the total number of cells administered to animals in the 5 × 10^6^ CAR-T20 cell group. The survival time was observed for 112 days following CAR-T20 administration. Approximately100 μL of 3 mg D-luciferin (J&K Scientific, 30 mg/mL dissolved in 9 mg/mL NaHCO_3_) was intraperitoneally administered to each mouse by intraperitoneal injection, and the optical intensity of Raji-Luc cells in each animal were determined using IVIS Lumina III (Perkin Elmer). Luminescence intensity was captured at 1, 3, 6, 9, 13, 16, 21,28,35,42,49,56,63,70,77,84,91,98,105 and 112 days following the administration of CAR-T20. Clinical symptoms were observed every day, while animal body mass and Raji-Luc bioluminescence intensity were measured every week.

#### In vivo* proliferation and antitumor effects of CAR-T20*

As blood collection has an impact on the animal physical condition and survival time, and the tissues/organs could only be collected during the necropsy, another 144 male NSG mice were employed and administered Raji-Luc cells (5 × 10^5^ cells per mouse, except those in the control group) prior to CAR-T20 dosing. These animals were randomized into 6 groups, which were the control group (non-tumor-bearing), buffer group, T-cell group, CAR-T20 1 × 10^6^ group, CAR-T20 3 × 10^6^ group and CAR-T20 10 × 10^6^ group, 6 in each group based on their bioluminescence intensities. Approximately 96 h after xenografting, animals were administered with buffer (both the control group and the buffer group), T cells (20 × 10^6^) and CAR-T20 cells (at dosages of 1 × 10^6^, 3 × 10^6^ and 10 × 10^6^). The dosages of regular CAR-T20 cells were estimated based on the proportion of CAR^+^ T cells (49.08%) in the batch used. The T-cell dosage was equivalent to the total number of cells administered to animals in the 10 × 10^6^ CAR-T20 cell group.

Peripheral blood of animals was collected at 1, 7, 14, 21, 28, 42, 56 and 90 days post-CAR-T-cell administration, and the serum was separated by centrifugation at 5000 rpm for 10 min at room temperature. Human cytokines (IL-2, IL-10, IFN-γ, and TNF, human Th1/Th2 cytokine cytometric bead array, Biosciences, Cat. No: 551809) and murine cytokines (TNF and IL-6, mouse Th1/Th2 cytometric bead array, Biosciences, Cat. No: 551287) were detected using flow cytometry (FACS Aria III, BD Biosciences, U.S.A).

Animals were anesthetized (by isoflurane inhalation) and sacrificed at 14, 28, 56 and 90 days post-CAR-T-cell administration. Serum was biochemically analyzed using a Hitachi 7180 Automatic Biochemical Analyzer (Tokyo, Japan) for parameters including glutamic-pyruvic transaminase (ALT), aspartic acid aminotransferase (AST), alkaline phosphatase (ALP), creatine phosphokinase (CK), lactic dehydrogenase (LDH), total protein (TP), and albumin (ALB). Peripheral blood was collected from the animals, and total T cells (Ph. PE-Cys Mouse Anti-Human CD45, BD Biosciences, Cat. No: 2138102; Ph.PerCP Mouse Anti-Human CD3, BD Biosciences, Cat. No: 0030910), CAR^+^ T cells (Human CD20 His Tag, Invitrogen, Cat. No: B327671; APC Anti-His Tag, BD Biosciences, Cat. No: 20CZF-2122) CD4^+^ cells(Ph.PE Mouse Anti-Human CD4, BD Biosciences, Cat. No: 7304679)and CD8^+^ cells (Ph.FITC Mouse Anti-Human CD8, BD Biosciences, Cat. No: 8270668) were analyzed by flow cytometry (FACSCalibur, BD Biosciences). Full necropsy was carried out, and tissues including the heart, liver, spleen, lungs, kidneys, brain, ovaries, uterus, testis, epididymis, abdominal skin, injection site, duodenum, jejunum, ileum, and bone marrow were fixed in 10% formalin. The tissues were stained with hemotoxylin and eosin for further histopathological examination.

#### Statistical analysis

All data are expressed as the mean ± standard deviation (SD), and the data from the number of the sample were indicated as “*n*”. For estimate the animal survival rate, Kaplan–Meier curves were generated. Other statistical analyses were carried out using one-way ANOVA, followed by Dunnett’s test for comparisons to the untreated or buffer groups. Figures were plotted using GraphPad Prism 8.0.1 (GraphPad Software, San Diego, CA, USA). Statistical significance was determined using SPSS (ver.12), and a significant difference is considered when *p* < 0.05.

## Results

### CAR-T20 targets and kills CD20 expressing cells in vitro

**Fig. 2:**
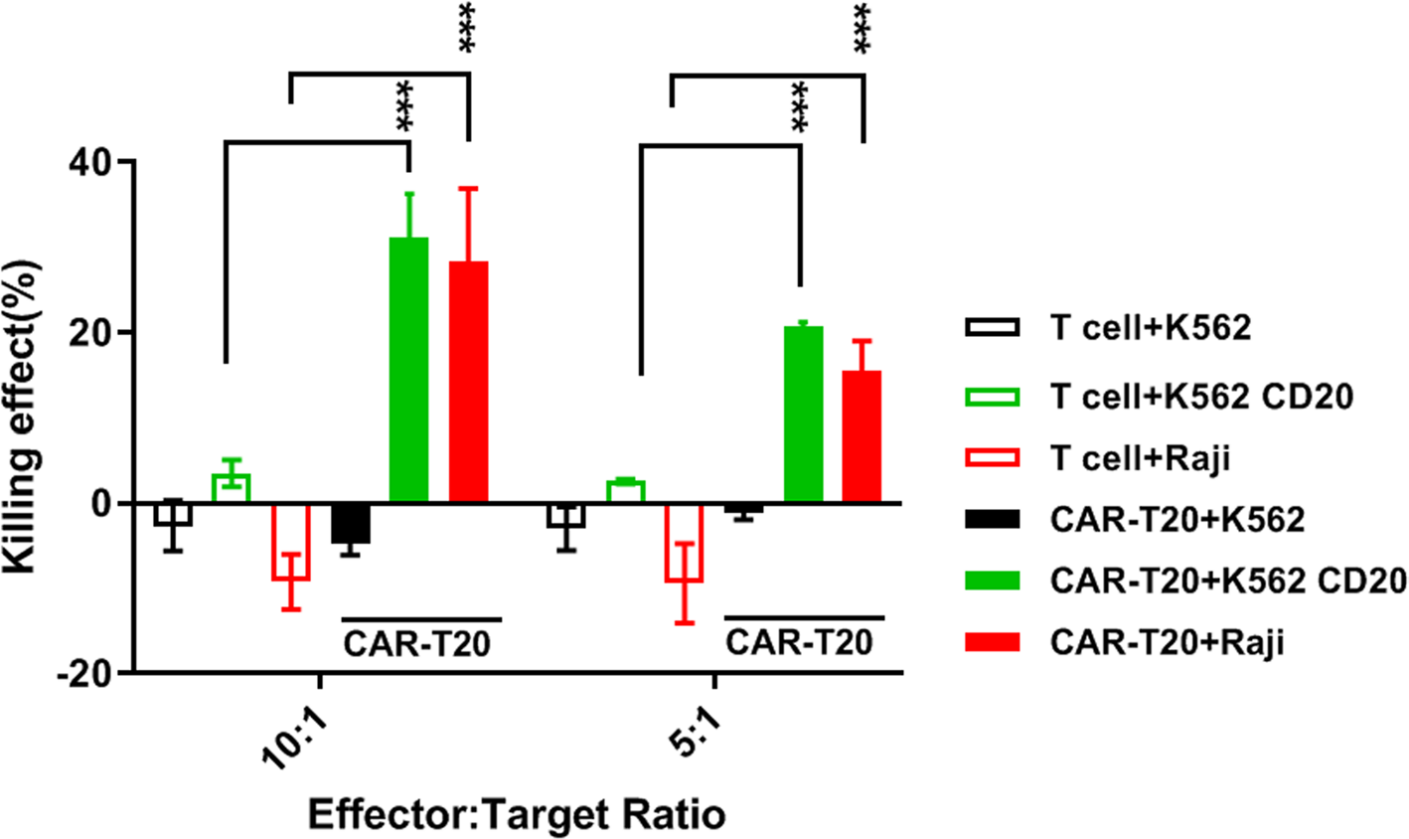
Targeted killing effects of CAR-T20 in vitro. The cell killing ration of CAR-T 20 to K562 cells, K562 cells transfected with CD20 (K562 CD20) and Raji cells at different effector: target ratios were shown. Data are shown as the mean ± SD (n = 6). ***p ≤ *0.001*

The CAR-T20 could recognize CD20 antigen and was proliferated significantly subsequent to the stimulation (Supplemental Fig. S1). To determine the specificity of CART-20 for CD20 antigen, an “effector/target” coculture system was adopted. T cells without CAR modification and CART-20 cells were co-cultured with either K562 lymphoblasts, K562 cells with CD20 expression (K562 CD20), or Raji cells with CD20 expression. As demonstrated (Fig. 2), the T cells showed no specific killing effect on the target cells, while the killing rates of CART-20 were 31.2% ± 12.5% and 20.6% ± 1.3% for the K562 CD20 cells and 28.3% ± 19.2% and 15.4% ± 8.7% for the Raji cells, at ratios of 10:1 and 5:1, respectively. The killing effect of CAR-T 20 on K562 CD20 and Raji cells was significantly different from that on T cells (p < 0.001), suggesting that CART-20 had a specific killing effect on CD20 expressing cells. CAR-T20 also exhibited similar killing effect in the cytokine release and CD107a expression analysis assays (Supplemental Fig. S2 and S3).

### CAR-T 20 effectively kills CD20 expressing cells and improves survival time in vivo

Abnormal clinical symptoms attributed to the receipt of Raji-Luc cells including roachback, piloerection, hind limb paralysis and reduced activity, were observed in the tumor-bearing animals (Table 1). Animals in the buffer group and T-cell group all died in the early stage of the experiment (within 15 days post-administration of buffer or T cells), while the animals administered CAR-T20 (at dosages of 1 × 10^6^, 2 × 10^6^ and 5 × 10^6^) developed symptoms at a later stage. Some animals administered 1 × 10^6^ or 2 × 10^6^ CAR-T20 developed roachback and piloerection within 14 days post-administration. For these animals, the above symptoms disappeared after 2 weeks and re-appeared at 1 month to 2 months post-administration. The re-appearance of roachback and piloerection in those who received 2 × 10^6^ CAR-T20 was much later than that in those who received 1 × 10^6^ CAR-T20. Most animals that received 5 × 10^6^ CAR-T20 cells showed no abnormalities during the experiment. The frequency and timing of the above symptoms were related to the dose of CAR-T20 administered.

**Table 1:** Summary of clinical symptoms

Group	Number of Animal	Number of animals without abnormal symptoms	Number of animals with abnormal symptoms			
			Roachback	Piloerection	Hind limb paralysis	Reduced activity
Buffer group	6	0	3	5	4	3
T-cell group	6	0	3	3	6	2
CAR-T20 1 × 10^6^ group	6	0	6	4	1	2
CAR-T20 2 × 10^6^ group	6	0	5	3	2	1
CAR-T20 5 × 10^6^ group	6	4	1	0	0	1

**Fig. 3:**
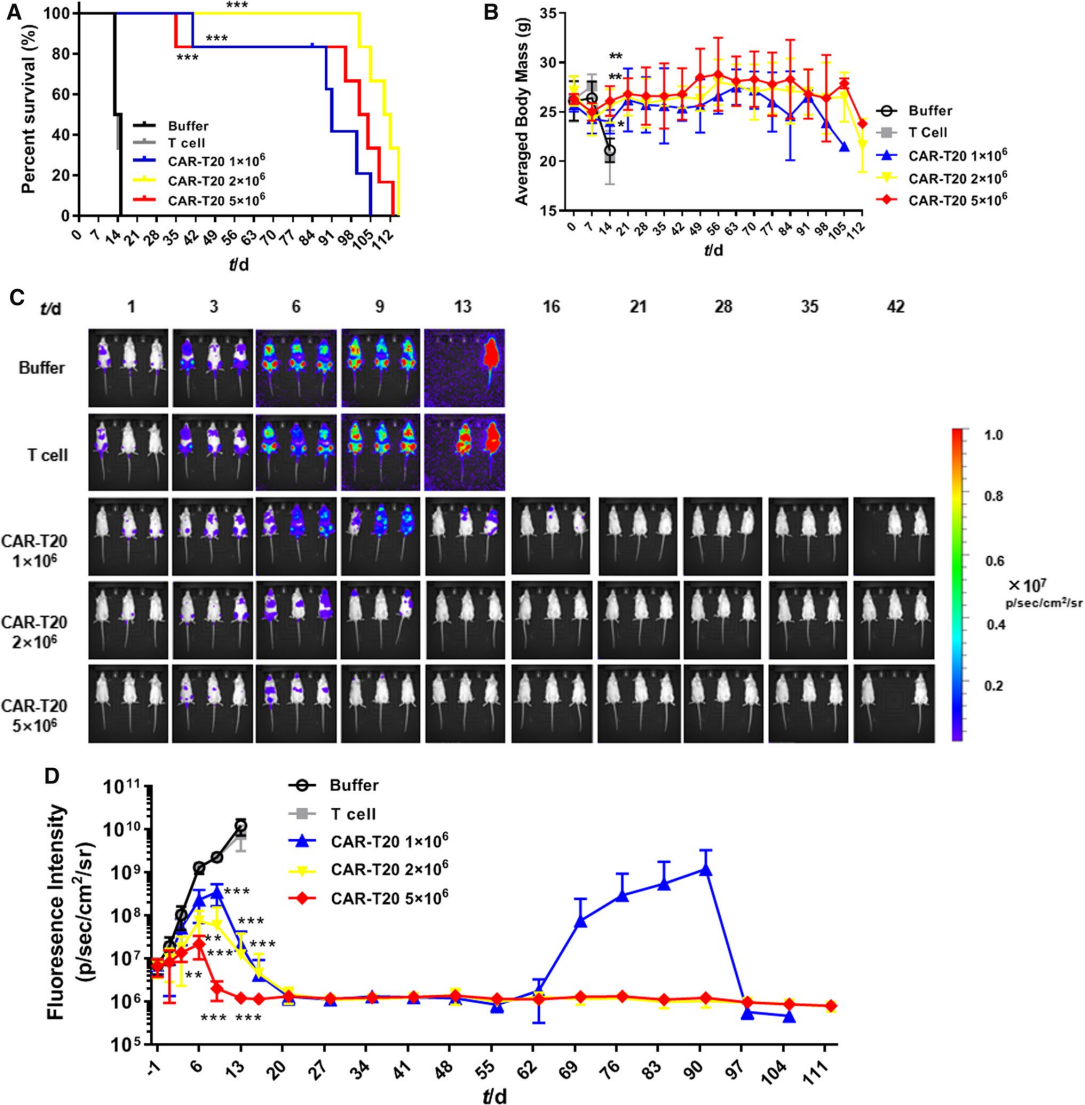
Effect of CAR-T 20 to the survival rate, body mass and tumor clearance in NSG. The NSG mice bearing Raji-Luc cells were administered with 1, 2 and 5 × 10 ^6^ CAR-T 20 respectively, and observed for over 16 weeks. **A** Mean survival rate; **B** Averaged body mass; **C** In vivo bioluminescence intensity for Raji-Luc clearance was visualized. **D** Averaged bioluminescence intensity. The CART-20 could prolong the survival rate, and decreased the overall intensities obviously the since 3 days after administered. Data are shown as the mean ± SD (n = 6). ***p ≤ *0.001*

The median survival times of animals administered Raji-Luc alone (buffer group), or in combination with T cells, and CAR-T20 (at dosages of 1 × 10^6^, 2 × 10^6^ and 5 × 10^6^) were 14 days, 13.5 days, 91 days, 111 days and 102.5 days (Fig. 3A). T cells failed to prolong the survival time of animals, and all animals died 15 days post-administration of 10 × 10^6^ T cells per animal. In contrast, CAR-T20 significantly prolonged the median survival time of tumor-bearing mice (*P* < 0.001), and 3, 6 and 5 animals in the CAR-T20 1 × 10^6^, 2 × 10^6^ and 5 × 10^6^ groups ( accounting for 50%, 100% and 83%, respectively) survived for over 90 days post-administration.

At the 2nd week post-administration, the average body weights of animals administered CAR-T20 (at dosages of 1 × 10^6^, 2 × 10^6^ and 5 × 10^6^) were significantly higher than those of the buffer group and T-cell group (*P* < 0.05, *P* < 0.01), with a slight dose correlation (Fig. 3B). There was no obvious weight loss in animals administered CAR-T20 until 90 days post-administration. These results suggested that CAR-T20 could ameliorate the weight loss induced by Raji-Luc cell proliferation.

The bioluminescence intensities for the proliferation of Raji-Luc cells in the mice are shown and summarized in Fig. 3C–D. The bioluminescence intensities in animals administered CART-20 were significantly lower (at dosages of 1 × 10^6^, 2 × 10^6^ and 5 × 10^6^) than those administered buffer (*P* < 0.05, *P* < 0.01) or T cells only (*P* < 0.05) from 6 days post-administration, and exhibited a dose–response relationship. On the 16th day after administration of buffer or T cells, all animals in buffer group and T-cell group died. At this timepoint, the average bioluminescence intensity of the animals administered CAR-T20 did not exceed 6 × 10^6^ p/sec/cm^2^/sr, which was lower than the level before CAR-T20 was administered after xenografting with Raji-Luc cells. For the animals dosed with 1 × 10^6^ CAR-T20, between the 21st to 63rd and 98th to 105th days post-administration of cells, the mean bioluminescence intensities of animals dosed with 1 × 10^6^ cells did not exceed 2 × 10^6^ p/sec/cm^2^/sr; however, between the 70th to 91st day post-administration of cells, the bioluminescence intensity of one animal increased significantly, resulting in an increase in the average bioluminescence intensity. This increase might indicate tumor recurrence. From the 21th to 112nd days after administration of cells, the mean bioluminescence intensity of animals in the middle- and high-dose groups did not exceed 1.5 × 10^6^ p/sec/cm^2^/sr.

### Proliferation of T cells and CAR-T cells in animals administered CAR-T20

The therapeutic effect of CAR-T20 on tumors expressing CD20 antigen has been demonstrated in Raji-Luc bearing mice at lower dose levels (1, 2, and 5 × 10^6^ CAR-T20 cells per mouse). To further understand the in vivo antitumor effect of CAR-T20, its lymphocyte distribution, cytokines and biochemical and histopathological characteristics were studied at a wider range of doses (1, 3, and 10 × 10^6^ CAR-T20 cells per mouse).

**Fig. 4:**
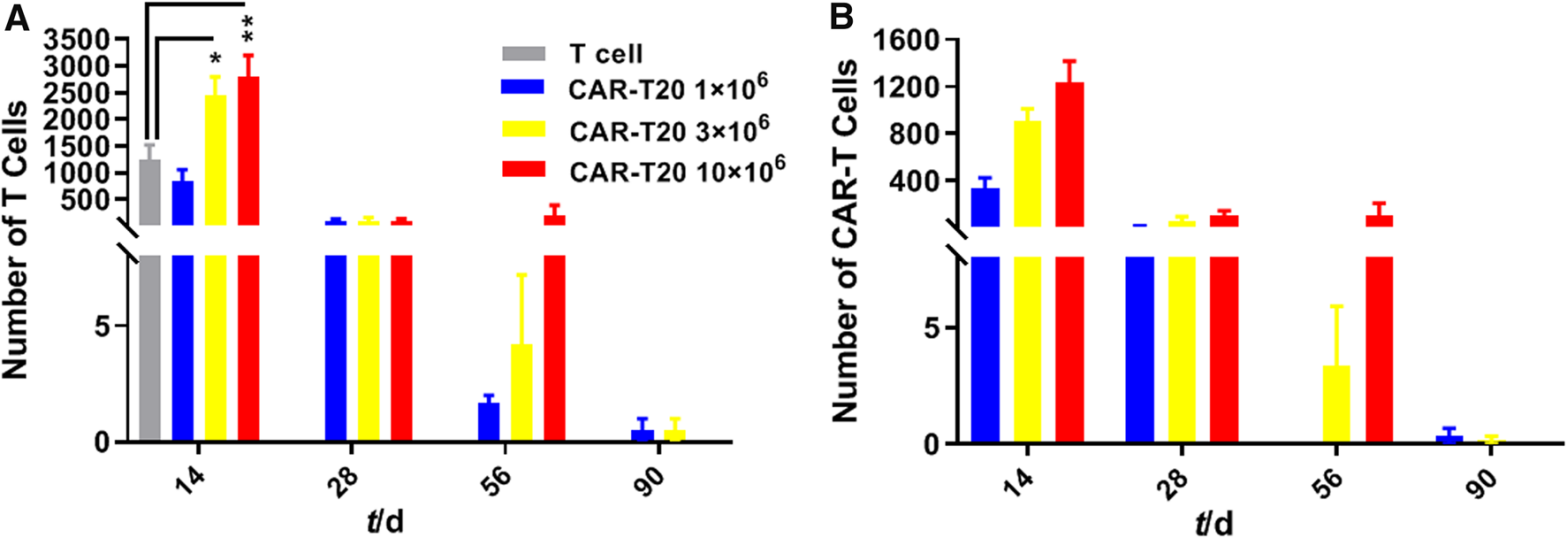
Proliferation of lymphocytes with T cells and CAR-T cells in animals administrated with CAR-T20. The NSG mice bearing Raji-Luc cells were administered with 1, 3 and 10 × 10 ^6^ CAR-T 20 respectively, and observed for 90 days. The counts of T cells (**A**) and CAR-T (**B**) cells were analyzed by flow cytometry method. The amounts of T cells and CAR-T in animals dosed with CAR-T20 reached the peak at 14 days post administration and decreased afterwards with a dose–response tendency. T cells in the T cell group increased predominantly from 14th to 28th day post administration. Data are shown as the mean ± SD (n = 6)

As shown in Table 2 and Fig. 4, 14 days post-administration, a large number of total T cells (62 ± 48 of which were identified as CD4^+^ and 1180 ± 756 of which were identified as CD8^+^) were detected in the T-cell group. At this time, a large number of total T cells and CAR-T cells were detected in animals administered CAR-T20, and they were mainly CD8^+^ cells. The number of CAR-T cells was proportional to the dose of CAR-T20. Compared with the T-cell group, both the CD4^+^ counts and CD8^+^ counts in the total T cells and CAR-T cells of the animals in the 3 × 10^6^ and 10 × 10^6^ dose groups were higher, and there was a significant difference (p < 0.05, p < 0.01). These results indicated that both T cells and CAR-T20 cells proliferated in tumor-bearing animals at this time, with CAR-T20 cells proliferating more than T cells.

**Table 2:** Number of human T cell and CAR-T cells in mice administered T cells or CAR-T20 (mean ± SD, n = 4 ~ 6)

Days post administration	Group	Total T cells		Total CAR-T cells	
		CD4^+^	CD8^+^	CD4^+^	CD8^+^
14	T cell group	62 ± 48	1180 ± 756	0 ± 0	0 ± 0
	CAR-T20 1 × 10^6^ group	121 ± 109	706 ± 527	52 ± 44	277 ± 231
	CAR-T20 3 × 10^6^ group	424 ± 176 **	2003 ± 674*	180 ± 89 **	676 ± 251 **
	CAR-T20 10 × 10^6^ group	846 ± 403 **	1615 ± 625	580 ± 227**	648 ± 235 **
28	CAR-T20 1 × 10^6^ group	10 ± 9	73 ± 101	6 ± 8	5 ± 4
	CAR-T20 3 × 10^6^ group	30 ± 37	65 ± 102	18 ± 21	49 ± 65
	CAR-T20 10 × 10^6^ group	67 ± 86	53 ± 69	55 ± 75	37 ± 52
56	CAR-T20 1 × 10^6^ group	1 ± 1	1 ± 1	0 ± 0	0 ± 0
	CAR-T20 3 × 10^6^ group	2 ± 4	2 ± 3	1 ± 3	2 ± 3
	CAR-T20 10 × 10^6^ group	82 ± 223	67 ± 187	42 ± 85	57 ± 132
90	CAR-T20 1 × 10^6^ group	0 ± 0	0 ± 0	0 ± 0	0 ± 0
	CAR-T20 3 × 10^6^ group	1 ± 2	0 ± 0	0 ± 1	0 ± 0
	CAR-T20 10 × 10^6^ group	0 ± 0	0 ± 0	0 ± 0	0 ± 0

Comparing with T cell group,*p < 0.05, **p < 0.01

At 28 days after administration of cells, only a small amount of total T cells could be detected in animals administered CAR-T20, and the mean total T cells in the 1, 3, and 10 × 10^6^ dose groups were 85, 99 and 142, respectively. CD4^+^ cells were dominant in the 1 × 10^6^ and 3 × 10^6^ dose groups. Although only a small number of CAR-T cells could be detected in the samples from the animals administered CAR-T20, the number of CAR-T cells was proportional to the dose of CAR-T20 administered. There was no statistically significant difference in the CAR-T-cell count among the groups. No differences in total T cells or total CAR-T-cell counts were observed at 56-and 90-days post-administration.

In summary, the numbers of T cells and CAR-T cells in the animals administered CAR-T20 increased significantly at 14 days post-administration; from 28 to 90 days post-administration, the numbers of T cells and CAR-T cells in animals administered CAR-T20 were markedly reduced. The above changes were related to the targeting effect of CAR-T20 and the inhibition of Raji-Luc cell proliferation.

### Effects of CAR-T20 on the proinflammatory cytokine profile in mice

**Fig. 5:**
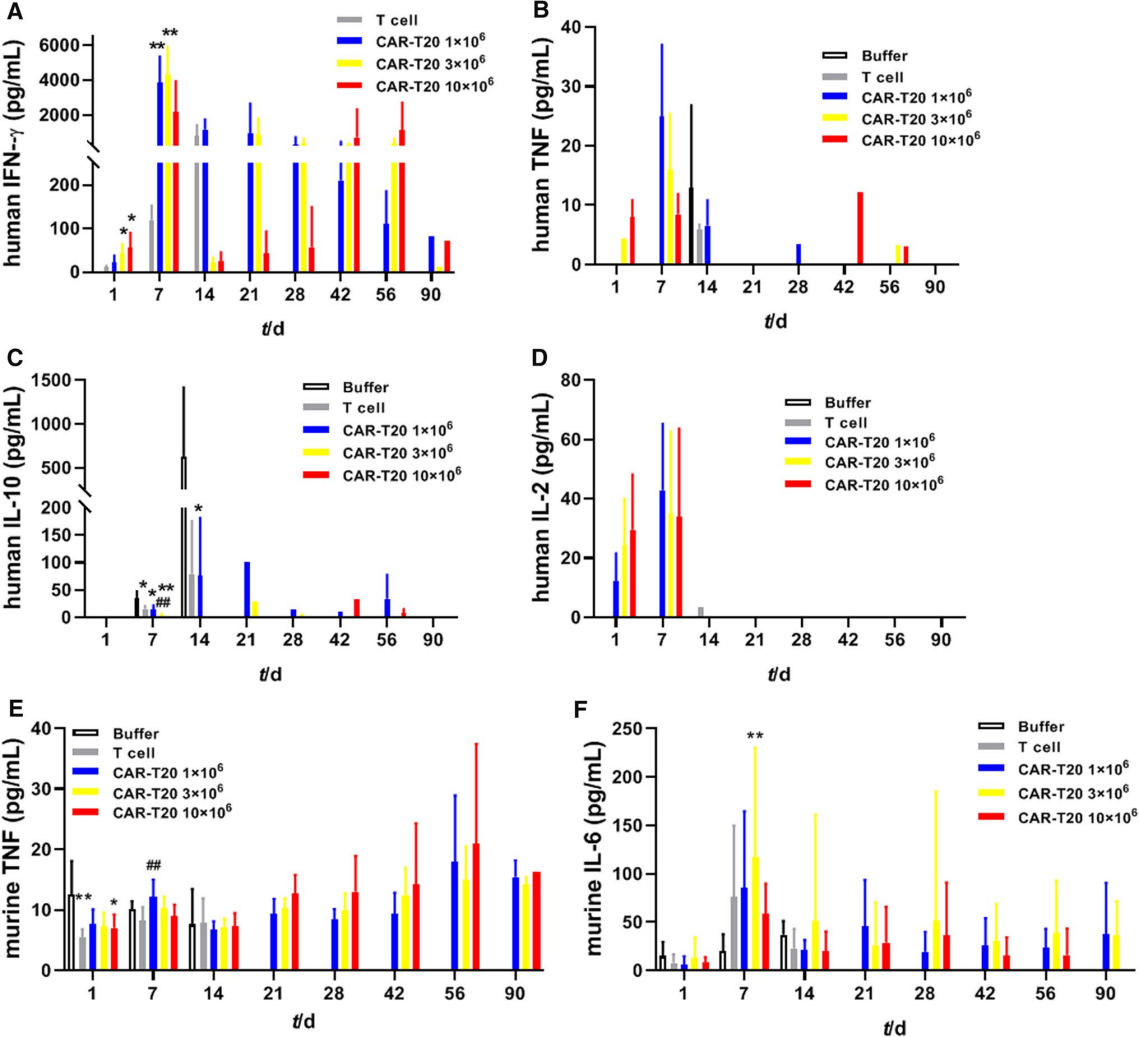
Cytokine profiles in NSG mice. The levels cytokines of human IFN-γ (**A**), human TNF (**B**), human IL-10 (**C**), human IL-2 (**D**), mouse TNF (**E**), and mouse IL-6 (**F**) in animals of each group at 1, 7, 14, 21, 28, 42, 56 and 90 days post administration are demonstrated. Comparing with the Buffer group, *p < 0.05, **p < *0.01;* comparing with the T cell group, *##p* < *0.01*

Both humanized and murine cytokines were examined using a flow cytometry method; see Fig. 5 for details. The average IFN-γ level of the T-cell group animals continued to increase after the cells were administered, reaching a peak (803.72 ± 618.64 pg/mL) at the 14th day post-administration. The averaged human IFN-γ levels of animals in the 1, 3, and 10 × 10^6^ dose groups peaked on the 7th day post-administration and decreased sharply with a dose‒response trend. However, the mean human IFN-γ level of animals in the 10 × 10^6^ dose group increased again at the 42nd day post-administration and reached a second peak (1153.38 ± 1551.91 pg/mL) at the 56th day. A small amount of human TNF was detected at different time points in the T-cell group and the CAR-T20 groups. In the CAR-T20 groups, human TNF level peaked the 7th day post-administration. In the buffer group, human IL-10 level peaked (626.85 ± 789.85 pg/mL) the 14 days post-administration, and the increase in human IL-10 was related to the proliferation of Raji-Luc cells in mice. The levels of human IL-10 in all CAR-T20 groups were lower than those in the buffer group and T-cell group over time, and there was a dose‒response relationship. Only a small amount of human IL-2 was detected within 14 days post-administration, and there was no statistically significant difference among the groups at different time points.

Murine TNF was detected at all times. On the 1st day post-administration, murine TNF levels in the T-cell group and the 10×10^6^ dose group were lower than those in the buffer group, and there was a significant difference (p<0.05, p<0.01). On the 7th day post-administration, the mean murine TNF level in the 1×10^6^ dose group was significantly higher than that in the T-cell group (p<0.05, p<0.01). The murine IL-6 level of animals in the buffer group continued to increase and peaked (36.92 ± 14.25 pg/mL) on the 14th day after administration. The murine IL-6 level of the animals in the T-cell group peaked (75.80 ± 73.92 pg/mL) on the 7th day post-administration and subsequently decreased, while the mean value remained above 20 pg/mL. Murine IL-6 levels of animals in the CAR-T20 1, 3 and 10×10^6^ dose groups peaked at the 7th day post-administration and decreased afterward.

### Effects of CAR-T20 on the serum biochemistry indices of tumor-bearing mice

**Fig. 6:**
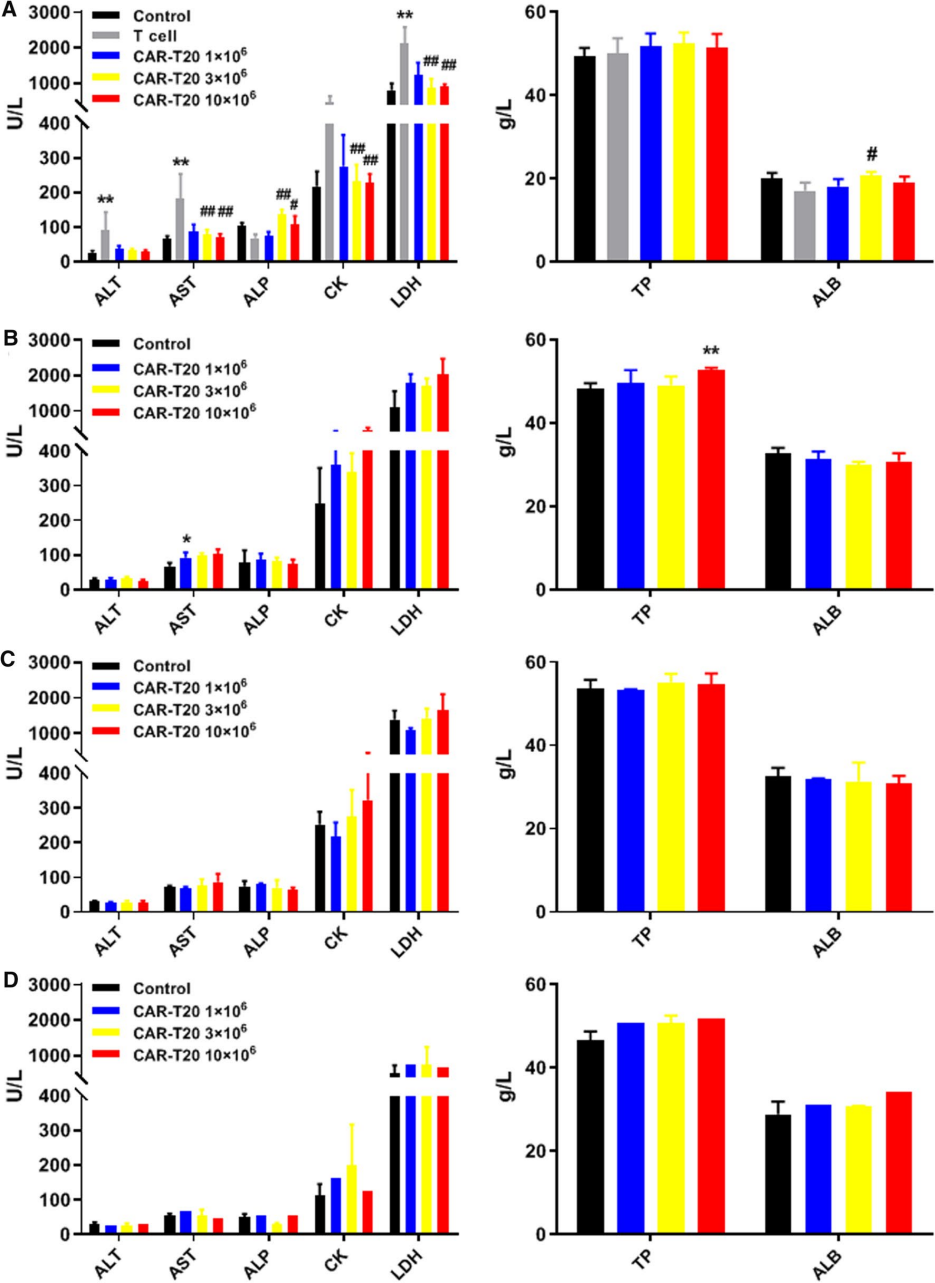
Effects of CAR-T20 on serum biochemistry indexes. The values ALT, AST, ALP, CK, LDH, TP and ALB at 14 days (**A**), 28 days (**B**), 56 days(**C**) and 90 days (**D**) after administration were shown. CAR-T20 mitigated the effects of Raji-Luc cells on liver function in mice and improved the malnutrition due to tumor burden. Data are shown as the mean ± SD (n = 6). Comparing with the Buffer group, *p < 0.05, **p < *0.01;* comparing with the T cell group, *#p* < *0.05, ##p* < *0.01*

Clinical chemistry indices were analyzed as shown in Fig. 6. At the 2nd week post-administration, the average ALT, AST and LDH levels of the animals in the T-cell group were significantly higher than those of the animals in the untreated group (*P* < 0.01), while the mean ALT, AST, CK and LDH levels of animals in the CAR-T20 3 × 10^6^ and 10 × 10^6^ dose groups were significantly lower than those of the animals in the buffer group (*P* < 0.01), suggesting that CAR-T20 alleviated Raji-Luc-related liver burden. In addition, the ALP level in animals administered CAR-T20 at 3 × 10^6^ and 10 × 10^6^ was significantly higher than that in the T-cell group (*P* < 0.05, *P* < 0.01), and the ALB level in animals administered CAR-T20 at 3 × 10^6^ was significantly higher than that in the T-cell group (*P* < 0.05). The reduction in ALP and ALB may be related to wasting and malnutrition caused by tumor burden. In general, the mean levels of biochemistry indices of animals administered CAR-T20 were closer to those of the animals in the control group (non-tumor-bearing group).

#### Histopathological changes

**Fig.7:**
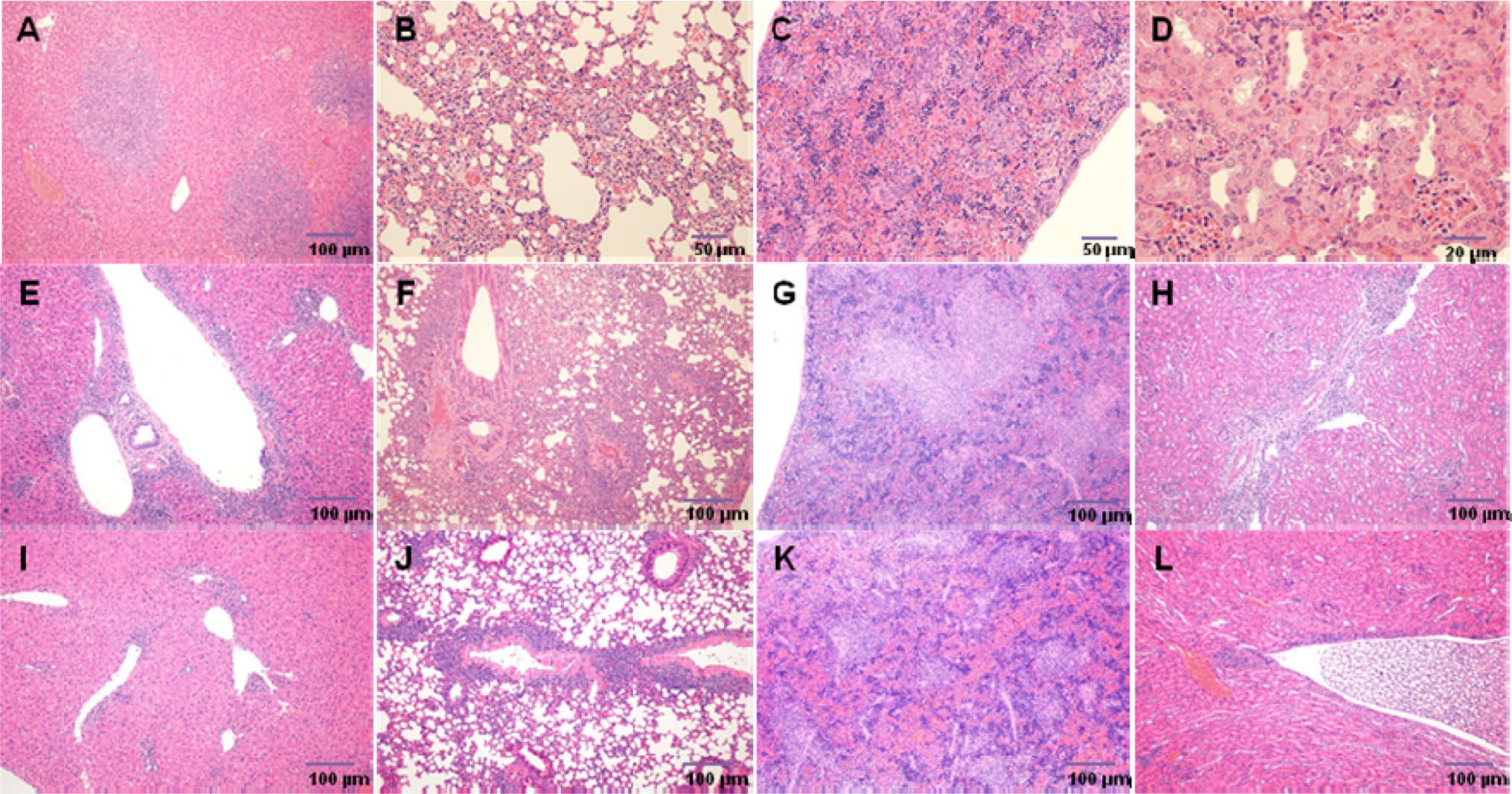
Histopathological changes of CAR-T20 in tumor-bearing animals (HE staining). **A** Buffer group, liver, lymphoma (100 ×); **B** Buffer group, lungs, lymphoma (200 ×); **C** Buffer group, spleen, lymphoma (200 ×); D Buffer group, kidney, lymphoma (400 ×); **E** T cell group, liver, mild mixed cell aggregation, very mild single hepatocyte necrosis, lymphoma (100 ×); **F** T cell group, lungs, moderate mixed cell aggregates, lymphoma (100 ×); **G** T cell group, spleen, mild mixed cell aggregation, lymphoma (100 ×); **H** T cell group, kidney, mildly mixed cell aggregation (100 ×); **I**T cell group, liver, mildly mixed cell aggregation (100 ×); **J** T cell group, lungs, moderate mixed cell aggregates, lymphoma (100 ×); **K** T cell group, spleen, mildly mixed cell aggregation (100 ×); **L** T cell group, kidney, very mild mixed cell aggregation (100 ×)

The histopathological changes revealed the in vivo pharmacological process of CAR-T20 in tumor-bearing mice, and the incidences of lymphoma are summarized in Table 3. Representative images are shown in Fig. 7. By the end of the 2nd week post-administration, the incidences of lymphoma in various tissues of the CAR-T20 3 × 10^6^ and 10 × 10^6^ dose groups were lower than those in the buffer group and T-cell group. By the end of the 4th week post-administration, no tumor was found in the 10 × 10^6^ dose group, and there was a dose‒response relationship of the tumor incidences in animals administered CAR-T20. These were believed to be the pharmacodynamic effects of CART20. In addition, the mixed cell aggregation (proliferation of T cells or CAR-T20 cells and related immune response cells, mainly mononuclear macrophages) in various tissues of the animals among the T-cell group and CAR-T20 1, 3 and 10 × 10^6^ dose groups was similar at the 2nd week post-administration, while the incidence and degree of mixed cell aggregation in multiple tissues in the T-cell group were higher than those in the CAR-T20 1, 3 and 10 × 10^6^ dose groups. These results suggest that the effect of CAR-T20 in the treatment of tumors is superior to that of T cells under the same degree of cell targeted proliferation during this period. The mixed cell aggregation in tissues in animals administered CAR-T20 was alleviated along with the tumor being cured, indicating the targeting effect of CAR-T20.

**Table 3:** Lymphoma incidences in different tissues of tumor-bearing mice

**Tissues**			**Lymphoma Incidence (%)**									
			**Heart**	**Lungs**	**Liver**	**Kidneys**	**Spleen**	**Spinal cord**	**Bone**	**Bone marrow**	**Aorta**	**Brain**
2 weeks post administration	Control		0	0	0	0	0	0	0	0	0	0
	Buffer		33	100	100	67	100	17	100	100	0	100
	T cell		0	100	100	100	100	17	100	100	17	67
	CAR-T20	1×10^6^	0	50	100	50	50	0	100	100	0	67
		3×10^6^	0	50	17	17	0	0	0	17	0	0
		10×10^6^	0	0	17	0	17	0	17	17	0	0
4 weeks post administration	Control		0	0	0	0	0	0	0	0	0	0
	CAR-T20	1×10^6^	0	0	17	0	17	0	0	33	0	17
		3×10^6^	0	0	0	0	0	0	17	17	0	0
		10×10^6^	0	0	0	0	0	0	0	0	0	0
8 weeks post administration	Control		0	0	0	0	0	0	0	0	0	0
	CAR-T20	1×10^6^	0	17	33	17	17	0	0	0	0	0
		3×10^6^	0	33	17	17	17	0	0	0	0	0
		10×10^6^	0	17	0	17	0	0	0	0	0	0
3 months post administration	Control		0	0	0	0	0	0	0	0	0	0
	CAR-T20	1×10^6^	0	0	33	0	0	0	0	0	0	0
		3×10^6^	0	0	0	0	0	0	0	0	0	0
		10×10^6^	0	0	0	0	0	0	0	0	0	0

By the end of the 8th week post-administration, the incidence of lymphoma in animals administered CAR-T20 had increased slightly, and the mixed cell aggregation was aggravated in these animals as well. These results suggest the possibility of tumor recurrence and the subsequent activation of CAR-T20 cells during this period. By the end of 90 days post-administration, there was no lymphoma and no mixed cell aggregation found in the survivors of the CAR-T20 3 and 10 × 10^6^ dose groups, indicating that they had been cured.

## Discussion

Chemotherapy has always been the mainstay of treatment for NHL, but it is not effective for all NHL subtypes, and a large proportion of patients are prone to relapse after standard chemotherapy regimens. The advent of immunotherapy has greatly improved NHL treatment. Phosphorylation of CD20 has been reported to be significantly higher in proliferating malignant B cells than in normal B cells [26] Rituximab is the first approved monoclonal antibody targeting CD20, which has a preferable therapeutic effect on almost all CD20^+^ B-cell NHL cases and has quickly become the first-line treatment for diffuse large B-cell lymphoma, follicular lymphoma and chronic lymphocytic leukemia [27]. However, approximately 40 ~ 50% of patients experienced drug resistance or relapse after treatment. As NHL progresses rapidly after relapse, the median survival time is only half a year [28]. CAR-T-cell therapy is the first FDA-approved cell therapy for the treatment of NHL. The patient's T cells are isolated and genetically modified to express CAR using genetic engineering technology and are then re-infused into the patient after in vitro screening and expansion. Antibodies on the surface of CAR-T cells can specifically recognize tumor cells and activate downstream related signaling pathways to kill tumor cells [29]. The mechanism through which CAR-T cells kill tumor cells includes releasing perforin and granzyme to induce tumor cell lysis, mediating tumor cell apoptosis through the Fas-FasL pathway, and secreting cytokines (e.g., IFN and interleukin) to the tumor microenvironment, thereby inhibiting tumor cell proliferation [30]. The advantages of targeting CD20 antigen have been well-demonstrated. Chu et al. reported that the combination of romidepsin, a histone deacetylase inhibitor, and anti-CD20 CAR-modified expanded peripheral blood natural killer (exPBNK) cells could significantly reduce tumor burden and enhance survival in humanized BL xenografted NSG mice [31]. In addition, there is feasibility of CAR-T cell that targets CD20 therapy after failure of CD20 antibody (e.g., rituximab) regimens. The sequence of SCFV targeting CD20 used in CAR-T20 recognizes a different epitope than rituximab. Experimental data have shown that CD20-CAR-T cells derived from scFv of the same origin as CAR-T20 could still efficiently expand in the presence of rituximab, and CD20-CAR-T cells can still recognize and lyse cells expressing low levels of CD20 antigen [32]. Although combination rituximab is a common treatment option for NHL, anti-CD20-CAR-T therapy remains effective after treatment failure or relapse: the overall response rate after R/R B-NHL treatment was 100% in 15 patients, with the best complete response rate of 80%. None of the patients developed grade 4 cytokine release syndrome, and only one patient developed immune effector cell-associated neurotoxicity syndrome [33].

In this study, we constructed CAR-T cells targeting the CD20 antigen for NHL therapy and verified the high killing rate of CAR-T20 on K652-expressing CD20 and Raji cells in an in vitro coculture system. Raji cells are lymphoblasts expressing CD19, CD20 and CD22 at high levels. Raji cells can generate lymphomas in multiple tissues in immunodeficient mice and are suitable for the study of the efficacy of different NHL-targeted drugs [34]. An in vivo study in tumor-bearing mice further indicated that CAR-T20 at a dose of 1 × 10^6^ per mouse or above effectively prolonged the median survival time from 14 days to more than 3 months, inhibited the proliferation of Raji cells in mice, and alleviated the clinical manifestations and weight loss caused by the Raji-Luc cell load. CAR-T20 at a dose of 2 × 10^6^ per mouse or above inhibited the proliferation of Raji cells in mice for up to 111 days post-administration (115 days after tumor xenografting) without recurrence, which is superior to similar CAR-T products reported in the literature [35]. As shown in the biochemistry data, CAR-T20 alleviated the effects of Raji-Luc cells on liver function in mice and improved the overall status and malnutrition caused by tumor burden. The efficacy of CAR-T20 was further confirmed by histopathological examination. On the 14th, 28th and 56th days post-administration, the incidences of xenografted tumors in organs/tissues were effectively reduced. Eight animals administered CAR-T20 survived until 90 days post-administration, and none of them were found to have lymphoma.

On the 14th day post-administration, the numbers of T cells and CAR-T cells in the animals administered CAR-T20 increased significantly, while they dramatically decreased from the 56th to 90th day post-administration. These results suggested that CAR-T20 cells are capable of targeting Raji-Luc cells in vivo. It is notable that the T cells were dominated by CD8^+^ T cells at 14 days post-administration, when the Raji cells were markedly proliferated. However, the numbers of CD4^+^ and CD8^+^ T cells tended to be similar in animals administered 10 × 10^6^ CAR-T20 at 28 days and 56 days post-administration, as the Raji cells were barely detected by that time. Differences in T-cell subtypes and the function of memory and effector T cells were found to be important factors in CAR-T-cell immunotherapy [36]. Both CD8^+^ and CD4^+^ subsets play synergistic roles in antitumor activities, and as demonstrated in patients with B-cell non-Hodgkin lymphoma [37] and in a mouse model [38], the high anticancer activity of CAR-T cells was correlated with a ratio of CD4^+^ to CD8^+^ T cells of 1:1. It was suggested that CAR-T cells containing polyfunctional T-cell subsets could deploy multiple immune programs represented by cytokines and chemokines, including IFN-γ, interleukin, and macrophage inflammatory proteins [36].

In addition, the levels of human and murine cytokines were monitored throughout the entire study period. CAR-T20 cells increased the level of human IFN-γ and decreased the level of IL-10 in tumor-bearing mice, both of which are related to the tumor-killing effect of CAR-T20 cells [24, 39]. For the CAR-T20 groups, the tumors were largely diminished at 14 days post-administration, and the levels of human IFN-γ and IL-10 in the tumor-bearing mice were also significantly reduced. Both T cells and CAR-T20 cells resulted in a slight increase in murine TNF levels, while tumor burden, T cells and CAR-T20 cells could also introduce a mild increase in murine IL-6. The above changes were the most obvious at 7 days post-administration and lasted until 90 days after administration of cells, suggesting that there was a sustained immune response in mice. Cytokine storm is the top concern for risk in the clinical application of CAR-T cells [40]. However, due to the limitation of immunodeficient animals, our study was not able to adequately evaluate the presence of cytokine storms in CAR-T cells. Histopathological examination revealed that the cause of death in some animals in the T-cell group and CAR-T20 groups may be related to GvHD or an excessive immune response caused by T-cell administration, which manifested as an increase in the number of bone marrow cells, mainly granulocytes [41].

The clinical research data of CAR-T20 has demonstrated its safety and effectiveness [33]. As results, all enrolled B-NHL patients who were previously R/R to rituximab achieved different degrees of clinical response with tolerable toxicities. The clinical safety and effectiveness of our CAR-T target on CD19 were also reported [42]. Although the difference on the indications, both are tolerable to the patients. As "live" drugs, cell therapy products could sustainably expand and target in particular tissues/organs in vivo. Many CAR-T therapeutic products are currently under development, and appropriate pharmacodynamic should be carried out in animal models to ensure the efficacy of clinical use. We demonstrated that our data has a good correlation with clinical trial, and non-clinical data obtained from this study mode could promote the clinical transformation of CAR-T products.

## Conclusion

Many CAR-T products have been developed for clinical purposes, but there is currently a lack of detailed and specific preclinical pharmacodynamic research scheme. To date, only a few studies on the efficacy of CD20-targeted CAR-T products in tumor-bearing animals have been reported. Our study systematically investigated the pharmacodynamic effects of targeting CD20 using in vitro and in vivo models. The effective dose of CAR-T20 in mice starts from 1 × 10^6^ per mouse, equivalent to a clinical dose of 5 × 10^6^/kg (the body mass of a mouse is taken as 0.02 kg, and the body surface area is taken as tenfold that of a human). As the CD20 antigen is more stable than the CD19, the probability of antigen relapse of CAR-T targeting at CD20 could be greatly reduced. However, CD20 antigen negative relapse could not be completely ruled out, and multiple-targeted CAR-T approaches could be future solutions [43]. This research laid the foundation for further clinical trials of CAR-T20 in R/R B-cell NHL patients and provided a reference for preclinical pharmacodynamic research of CAR-T cells for NHL treatment.

## Supplementary Information

Supplementary file 1 (DOCX 748 KB)

## Data Availability

The data used to support the findings of this study are included within the article and supplementary information file.
