# Supplementary material for: Pre-clinical efficacy of CD20-targeted chimeric antigen receptor T cells for non-Hodgkin's lymphoma
Source: Discov Oncol. 2022 Nov 9;13:122. doi: 10.1007/s12672-022-00588-w (PMC9646688; doi:10.1007/s12672-022-00588-w)
Supplement: Supplementary file 1 — (DOCX 748 KB) [file 12672_2022_588_MOESM1_ESM.docx]

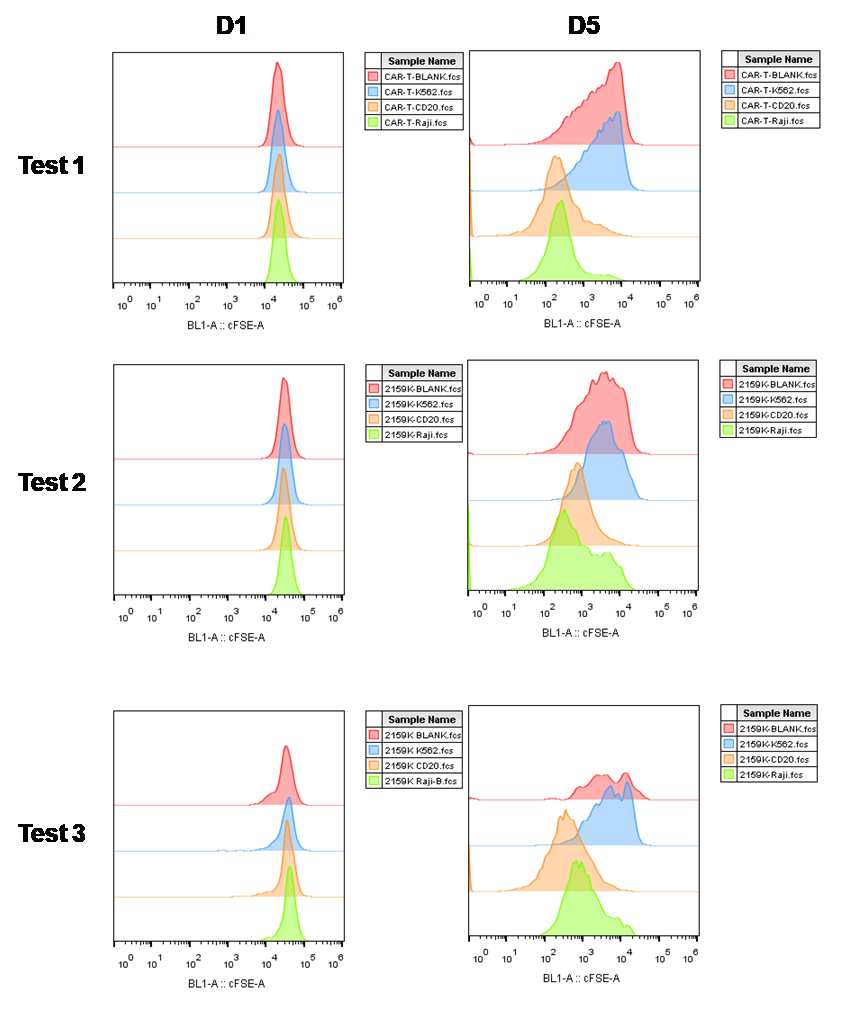


**Supplemental Fig 1. Specific Proliferation of CAR-T20 *in vitro.***

The CAR-T20 was labeled with CFSE, and the cell proliferation was detected at D1(left panel) and D5(right panel). The experiment was repeated for 3 times.

CAR-T cells have the ability to specifically recognize tumor cells with corresponding antigens. CAR-T cells stimulated by tumor antigens could activate and continuously proliferate to kill tumor cells. The proliferation ability of CAR-T cells after tumor cell stimulation is particularly important for the competition between CAR-T cells and tumor cells *in vivo*.

Fuorescent dye CFSE (also known as 5,6-carboxyfluorescein diacetate, succinimidyl ester), was used to label effector cells, and the effector cells: target cells were co-incubated at a ratio of 2:1 for 5 days. The level of CFSE was detected by flow cytometry at D1 and D5. As results, marked left shift of fluorescence were observed when CAR-T20 cells were co-incubated with antigen-positive target cells, indicating that CAR-T20 cells could recognize CD20 antigen and was proliferated significantly subsequent to the stimulation. In contrast, proliferation of CAR-T20 cells by CD20-negative K562 was not obvious.


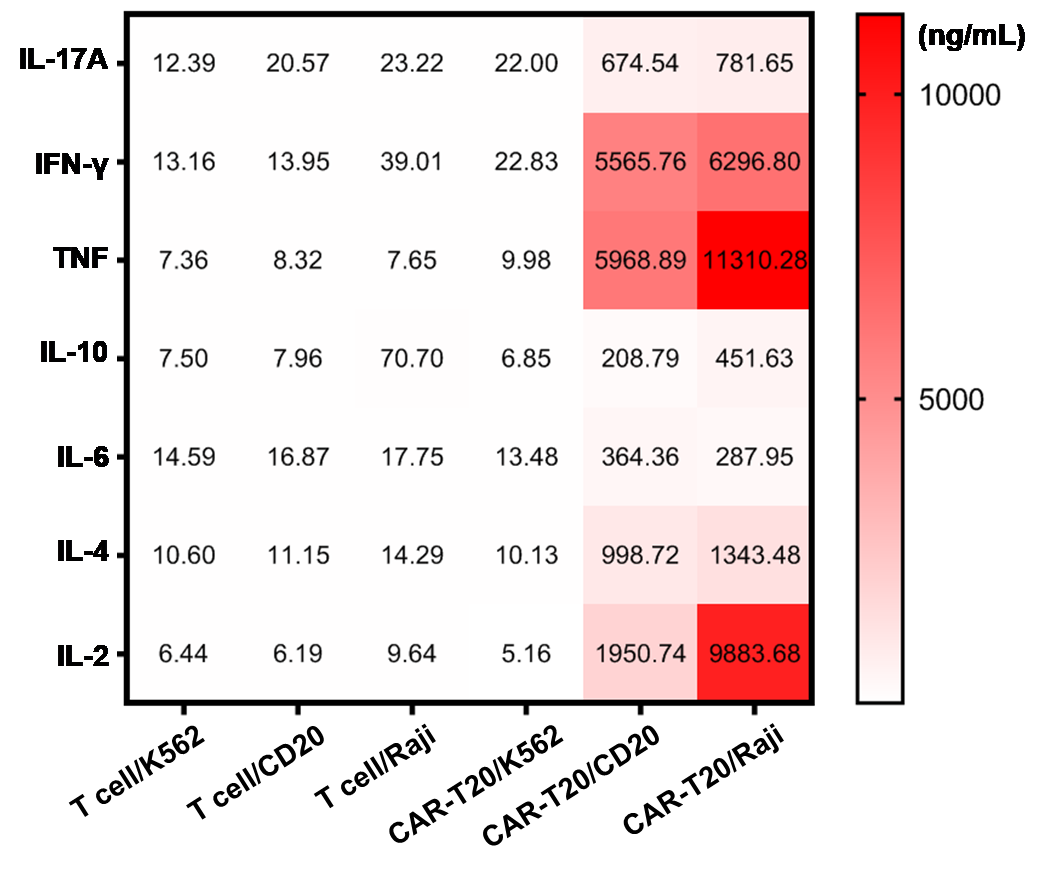


**Supplemental Fig 2. Cytokine release level of T cells and CAR-T20 *in vitro.***

T cells and CAR-T20 were co-incubated with target cells for 18 hours, and the levels of cytokines (including IL-2, IL-4, IL-6, IL-10, IL-17A, TNF-α and IFN-γ) were detected respectively.

CAR-T cells stimulated by tumor antigens could secrete a variety of cytokines, including IL-2, IL-6, IFN-γ, TNF- α, *etc.*, which reflects the killing capability of CAR-T cells.

Effector cells were co-incubated with target cells for 18 hours, the level of cytokine release in the supernatant was measured using a human cytokines CBA kit (IL-2, IL-10, IFN-γ, and TNF, human Th1/Th2 cytokine cytometric bead array, Biosciences, Cat. No: 551809). As results, multiple cytokines (including IL-2, IL-4, IL-6, IL-10, IL-17A, TNF-α and IFN-γ) were released at relatively higher levels, subsequent to the co-incubation of CAR-T20 and CD20 antigen or Raji cells. In contrast, the cytokines released in T cells co-incubation groups and CAR-T20/K562 co-incubation group are very low.


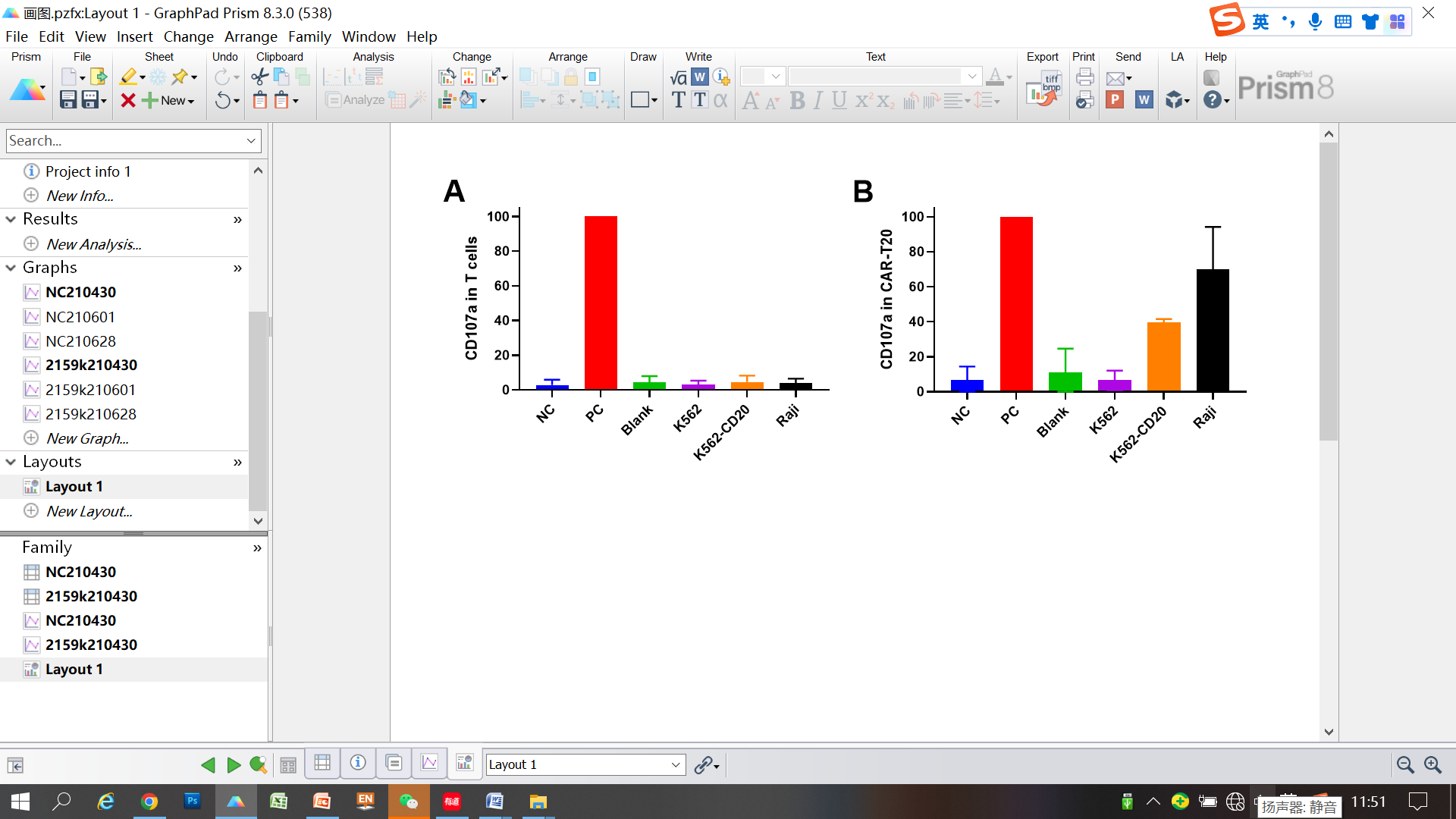


**Supplemental Fig 3. Expression level of CD107a *in vitro (n=3).***

Target cells were co-incubated with T cells (A) or CAR-T20 (B) overnight, and the expression levels of CD107a for cell were detected respectively.

CD107a (a type of lysosome-associated membrane protein-1), which is normally expressed on the surface of T cells, is a highly glycosylated protein that is commonly used in detecting cell-mediated cytotoxicity.

Effector cells and target cells were incubated overnight at a ratio of 5:1, and a positive control group was set in parallel (PMA). The expression level of CD107a molecule at CD20 was used to determine the killing efficacy of CAR-T cells. When co-incubated with antigen-positive target cells, The levels of CD107a in the CAR-T20 and CD20 or Raji co-incubation groups were much higher that of the negative control and the CAR-T20 and K562 co-incubation group; while the T cells were unable to increase the levels of CD107a. These indicated that CAR-T20 could recognize CD20 antigen and kill CD20 antigen-positive tumor cells specifically.

**
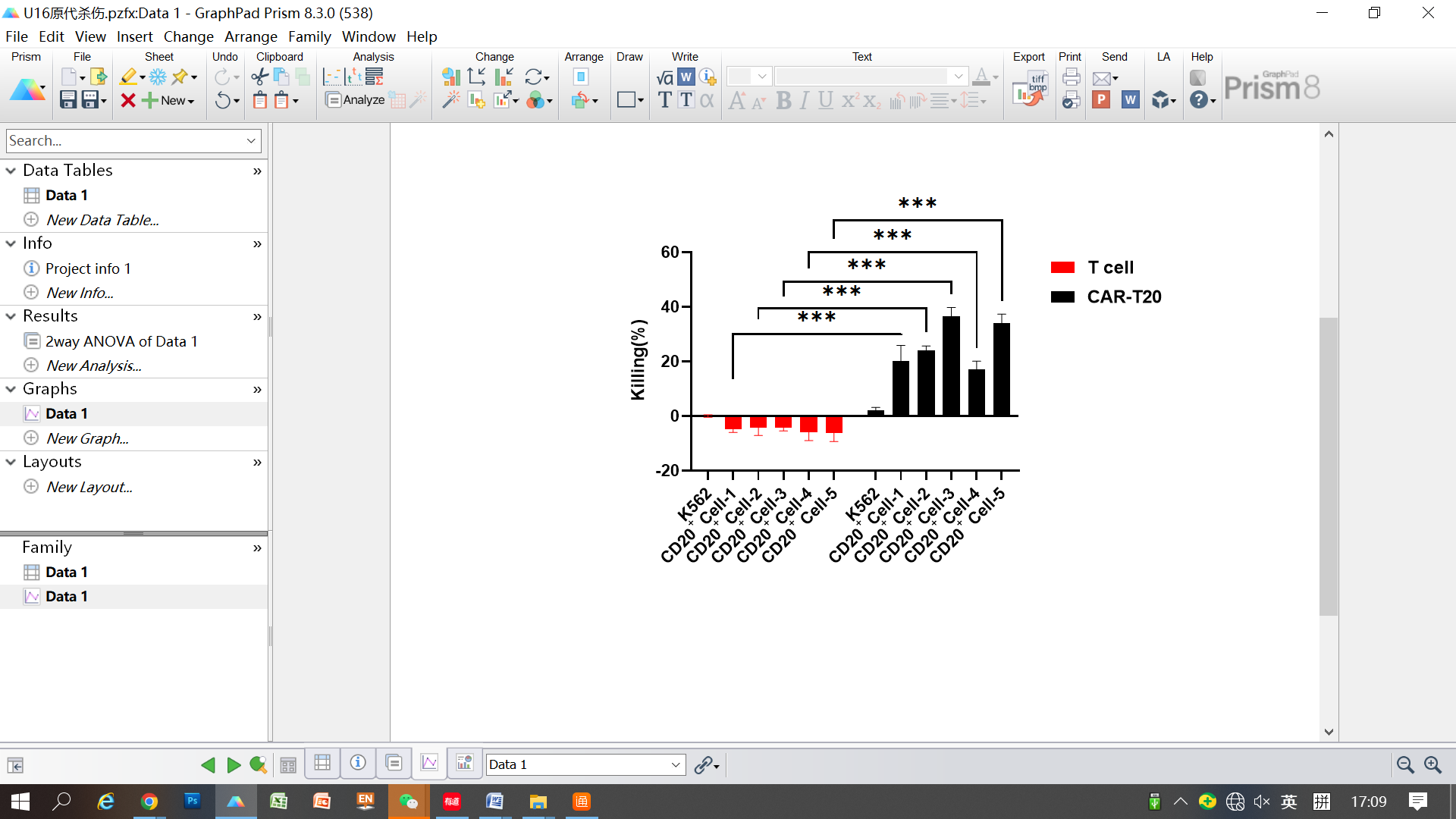
**

**Supplemental Fig 4. Killing effect of CAR-T20 on the CD20^+^ peripheral blood mononuclear cells *in vitro (n=3)*.**

Human peripheral blood mononuclear cells **(**PBMCs) from five donors were collected, and the CD20^+^ cells were sorted out using anti-CD20 magnetic beads. The CD20^+^ cells separated from different donors were named as CD20^+^ Cell-1, CD20^+^ Cell-2, CD20^+^ Cell-3, CD20^+^ Cell-4 and CD20^+^ Cell-5, respectively. Effector cells (T cells or CAR-T20 of about 5×10^6^) were co-incubated with the target cells (K562 cells or the primary CD20^+^ cells) for 24 h. The released level of lactate dehydrogenase of each sample was determined by a microplate reader at 490 nm, and the killing ratio of effector cells was calculated. As results, CAR-T20 showed killing effects to the PBMCs from all the donors at about 15%~40%, while the T cell was not able to kill the PBMCs (Student’s t-test, ***p<0.001). Both CAR-T20 and T cells had no killing effect to the K562 cells (CD20^-^). In summary, the CAR-T20 demonstrated specific killing effect on the human CD20^+^ primary cells.
